# Associations between long-term exposure to air pollution and kidney function utilizing electronic healthcare records: a cross-sectional study

**DOI:** 10.1186/s12940-024-01080-4

**Published:** 2024-04-23

**Authors:** David Dillon, Cavin Ward-Caviness, Abhijit V. Kshirsagar, Joshua Moyer, Joel Schwartz, Qian Di, Anne Weaver

**Affiliations:** 1 Center for Public Health and Environmental Assessment, United States Environmental Protection Agency, Research Triangle Park, NC USA; 2 Division of Nephrology and Hypertension, University of North Carolina School of Medicine, Chapel Hill, NC USA; 3 T.H. Chan School of Public Health, Harvard University, Boston, MA USA; 4 Research Center for Public Health, School of Medicine, Tsinghua University, Beijing, China

**Keywords:** Chronic kidney disease, Air pollution, Fine particulate matter, Ozone, Nitrogen dioxide, Electronic healthcare records

## Abstract

**Background:**

Chronic kidney disease (CKD) affects more than 38 million people in the United States, predominantly those over 65 years of age. While CKD etiology is complex, recent research suggests associations with environmental exposures.

**Methods:**

Our primary objective is to examine creatinine-based estimated glomerular filtration rate (eGFR_cr_) and diagnosis of CKD and potential associations with fine particulate matter (PM_2.5_), ozone (O_3_), and nitrogen dioxide (NO_2_) using a random sample of North Carolina electronic healthcare records (EHRs) from 2004 to 2016. We estimated eGFR_cr_ using the serum creatinine-based 2021 CKD-EPI equation. PM_2.5_ and NO_2_ data come from a hybrid model using 1 km^2^ grids and O_3_ data from 12 km^2^ CMAQ grids. Exposure concentrations were 1-year averages. We used linear mixed models to estimate eGFR_cr_ per IQR increase of pollutants. We used multiple logistic regression to estimate associations between pollutants and first appearance of CKD. We adjusted for patient sex, race, age, comorbidities, temporality, and 2010 census block group variables.

**Results:**

We found 44,872 serum creatinine measurements among 7,722 patients. An IQR increase in PM2.5 was associated with a 1.63 mL/min/1.73m^2^ (95% CI: -1.96, -1.31) reduction in eGFRcr, with O_3_ and NO_2_ showing positive associations. There were 1,015 patients identified with CKD through e-phenotyping and ICD codes. None of the environmental exposures were positively associated with a first-time measure of eGFR_cr_ < 60 mL/min/1.73m^2^. NO_2_ was inversely associated with a first-time diagnosis of CKD with aOR of 0.77 (95% CI: 0.66, 0.90).

**Conclusions:**

One-year average PM_2.5_ was associated with reduced eGFR_cr_, while O_3_ and NO_2_ were inversely associated. Neither PM_2.5_ or O_3_ were associated with a first-time identification of CKD, NO_2_ was inversely associated. We recommend future research examining the relationship between air pollution and impaired renal function.

**Supplementary Information:**

The online version contains supplementary material available at 10.1186/s12940-024-01080-4.

## Introduction

Chronic kidney disease (CKD) is a prevalent and growing health concern. Globally, CKD resulted in 1.2 million premature deaths in 2017, with an estimated prevalence of 697.5 million [1, 2]. Annual mortality is expected to increase 2.2–4.0 million by 2040 [2]. More than 38 million people in the United States live with CKD, most of whom are over the age of 65 [3, 4]. Following worldwide trends, the prevalence in the US is expected to increase in the coming decades; for those over 65 years of age, the prevalence is expected to increase by 37.8% by 2030. Older adults often experience higher levels of difficulty with both the successful management of CKD and, once the disease has progressed to end-stage kidney disease (ESKD), access to the resources necessary for kidney transplantation [5].

CKD is broadly defined by the presence of an estimated glomerular filtration rate (eGFR) of less than 60 mL/min per 1.73 m^2^, markers of kidney damage such as albuminuria or hematuria, or both for a duration of >  = 90 days, or the need for kidney replacement therapy [6]. Even a moderate decrease (eGFR of 59 to 30 mL/min per 1.73 m^2^) in kidney function increases the risk of hospitalization [7]. There are five stages of CKD, with stage 1 being the mildest and stage 5 indicated either severe impairment (eGFR < 15 mL/min per 1.73 m^2^) or kidney failure. Given the relatively mild symptoms of mild to moderate decreased kidney function, most individuals are not aware when they are in the first few stages of CKD. Prevalence of early CKD appears to be higher in females, but males progress more quickly through the disease stages and have a higher risk of mortality [8]. While standardized mortality rates for other non-communicable diseases such as cancer and cardiovascular disease have declined, CKD has not seen the same substantial decrease [9].

Most cases of CKD are caused by diabetes, hypertension, or a combination of both conditions, while other less common causes include primary glomerulonephritis, chronic tubulointerstitial nephritis, hereditary disease, secondary glomerulonephritis or vasculitis, etc. [10]. These issues are the manifestation of a combination of genetic, behavior, and environmental factors [11]. The mechanisms by which these diseases damage the kidneys over time include, but are not limited to, systemic/intraglomerular hypertension, glomerular hypertrophy, precipitation of intrarenal calcium phosphate, inflammation, and altered metabolism [10]. This chronic, consistent damage changes the overall architecture of the kidney, leading to scarring, and reduces their ability to function normally.

Recent research suggests environmental exposures as potential factors associated with the onset and progression of CKD in addition to these other factors [12]. Long-term exposure to air pollutants, specifically coarse particulate matter (PM_10_), fine particulate matter (PM_2.5_), and nitrogen dioxide (NO_2_), show a mixed, but overall, consistent relationship with low kidney function [13, 14]. It is possible that there is translocation of ultrafine particles directly into the bloodstream, oxidative stress responses, or changes in the ratios and total number of immune cells [15, 16]. Ozone (O_3_) may impact the kidneys as inhalation induces immunosuppressive and metabolic responses in the kidneys, heart, and liver [17]. Animal models suggest that inhalation of O_3_ alters gene expression in pathways involving inflammatory signaling, antioxidation, and endothelial function [18]. However, few studies have examined the effect of O_3_ on kidney disease in humans.

Our primary objective is to examine if airborne exposure to PM_2.5_, O_3_, or NO_2_ is associated with (1) reduced renal function as measured by serum creatinine estimated eGFR_cr_ or (2) a diagnosis of CKD in a random sample of patients from the University of North Carolina Healthcare System (UNCHCS).

## Methods

### Study population

We defined the sampling frame for this study to include patients with available electronic health records (EHRs) containing information on kidney health. Specifically, we include those with available serum creatinine laboratory test results and ICD codes relevant to CKD (available in Supplemental Table 1). We then separate this population into two distinct groups. The first group consists of all individuals with reported lab values for serum creatinine. We will use this group to analyze associations between air pollution and eGFR_cr_ continuously. The second group is restricted to patients with two measures of eGFR_cr_ < 60 mL/min per 1.73 m^2^ > 90 days apart and/or an ICD code indicating a physician diagnosis of CKD III-V. This group we consider as a positive case for CKD. These positive cases will be matched with controls for analysis.

To accomplish this, we utilize data from EHRs in the in Environmental Protection Agency’s Clinical and Archived Records Research for Environmental Studies (EPA CARES) [19, 20]. Our sampling frame from the EPA CARES population is a random sample of 19,989 individuals (504,406 unique visits), who were seen at a UNCHCS affiliated hospital or clinic from January 1st, 2004, to December 31st, 2016. Any participants with implausible demographic information (e.g., older than 110 years, BMI above 50, etc.) were removed prior to any analysis as it is likely these values were introduced during errors in entering information in electronic health records. Additionally, we removed any individuals who did not reside in North Carolina (*n* = 403). For both groups (continuous outcome and binary), we linked the 1-year average PM_2.5_, O_3_, and NO_2_ prior to the date of the (group 1) serum creatinine laboratory tests or (group 2) the second eGFR_cr_ value < 60 mL/min per 1.73 m^2^ or ICD code, whichever occurs earliest.

### Assessment of renal function

For the eGFR analyses, we use serum creatinine levels to assess kidney function as our first outcome. We estimated eGFR_cr_ using the 2021 CKD-EPI equation for serum creatinine:

$$\text{eGFR}_{\text{cr}} = 142 \times min(\text{S}_{\text{cr}}/\upkappa,\ 1)^{\upalpha} \times max(\text{S}_{\text{cr}}/\upkappa,\ 1)^{\text{-1.200}} \times \text{0.9938}^{\text{Age}} \times \text{1.012}\ [if\ female]$$

This equation was updated in 2021 to no longer include race in estimates of eGFR_cr_. Here, Scr is serum creatinine in mg/dL, κ is 0.7 for females and 0.9 for males, α is -0.241 for females and -0.302 for males, the min and max represent the minimum or maximum of the specified measurement or 1 [21]. We used the Tukey method to remove outliers, eliminating serum creatinine values more than 1.5 standard deviations above Q3 or those 1.5 standard deviations below Q1 before we calculate eGFR_cr_ [22]. Information on UACR and serum cystatin C were not included in this analysis as they were not available in the data. The analyses focused on eGFR_cr_ as a continuous outcome includes all recorded measures. Individuals may be included multiple times in the same dataset.

For our second outcome of interest, we designate an e-phenotype using similar methods described in previous research focused on kidney health utilizing EHRs [23, 24]. We consider a positive case of CKD stage III-V if a patient presents with two eGFR_cr_ measures < 60 mL/min per 1.73 m^2^ greater than 90 days apart or has an ICD code indicating physician diagnosis. If patient data contains both types of diagnoses, we take the earliest diagnostic date. If a patient only has eGFR_cr_ measures, we take the second measure as the diagnostic date. ICD-9 codes include 585.3 – 585.6 and ICD-10 codes include N-18.3 – N18.6. We use this method as many people living with CKD are unknowingly living with CKD and may not be diagnosed by a physician. Using similar methods, Paik et al. 2021 achieved positive predictive values > 80% [23]. One-year annual air pollutant averages for the preceding 365 days are linked to the exact serum creatinine laboratory date as exposures.

### Matching identified CKD cases and controls

For our CKD analyses, to ensure that our sampling was robust against bias, we matched each case to four controls (1:4) who were never diagnosed or identified as having CKD by e-phenotyping. We performed this matching using the ‘MatchIt’ package in RStudio. This package allowed for matching cases and controls based on designated input variables to produce more robust results with less sensitivity to assumptions. We matched according to propensity scores based on diagnosis date, age, race, and sex. For controls, who do not have a diagnosis date, we match on the closest hospital visit (Supplementary Fig. 2). We matched on the ‘optimal’ controls using the propensity score generating by matching variables. To ensure that we were not selecting matches from different geographic regions of the state, potentially introducing confounding, we compared cases and control percentages taken from the eight climate divisions of the state (see Supplementary information) [25]. This matching was only done for patients with identified CKD. We then calculate differences in dates between cases and controls to ensure that we are sampling from similar time frames.

### Exposure assessment

For PM_2.5_ and NO_2_ data, we used an ensemble model constructed by Di et al. that incorporates satellite aerosol measures, land-use regression, chemical transport models, and meteorological data [26]. This model incorporates three machine learning algorithms that predict pollutant concentrations in 1 × 1 km grids for the entirety of the contiguous Unites States. This model has been cross validated with an R^2^ of 0.89 (for the US Middle Atlantic Region) and shows accurate performance up to concentrations of approximately 60 µg/m^3^ or less [26]. The CARES patient data has the primary addresses of patients which we link to the appropriate 1 × 1 km grid. Where primary addresses were not successfully geocoded, we matched patients to the 1 × 1 km grid cell of the centroid of their primary residence ZIP code. O_3_ data come from the 12 km^2^ Community Multiscale Air Quality Modeling System (CMAQ) model; specifically, we use averaged 8-h maximum concentrations for O_3_ and averaged 24-h for NO_2_ [27]. CMAQ utilizes hourly measured pollutant data along with meteorological information to estimate pollutant concentrations at the census tract level. For all three pollutants, we estimate annual averages for all included patients.

### Covariates

We chose covariates based on previously published research examining associations between air pollution and renal function [28]. We include individual-level sociodemographic information of age, race (Caucasian, African American, other), and sex as factors. We created the ‘other’ race category as there were too few patients of other racial backgrounds that were not Caucasian or African American to include separately in models. Clinical diagnosis of both diabetes and hypertension were included in descriptive statistics based on ICD-9 and ICD-10 codes (250.x and E11.x for diabetes and 401.9 and I10 for primary hypertension) (full list of ICD codes available in Supplementary information). However, these were excluded from models as they are both likely mediators of kidney function and onset of CKD. Event-specific instances of these diseases (e.g., pregnancy induced hypertension) were not included in this analysis. We adjusted for the following 2010 census/2013 5-year ACS variables at the block group level: income, percent older housing (built before 1979), percent living in poverty, urbanicity, and percent of the population on public assistance, all as continuous covariates. Education (percent with a bachelor’s degree or higher) and median price of housing were included in descriptive statistics but excluded from final models due to high collinearity (r >|0.7|) with income. Lastly, climate zones (identified from climatechange.nc.gov) were included as regional adjustment for unmeasured factors that differ between regions in North Carolina as a factor in our models. Smoking status and body mass index (BMI) were not included in the main analyses as they were not recorded for a large portion of patients and only reported as secondary analyses.

### Statistical analyses

We analyzed associations between eGFR_cr_ and air pollutants using linear mixed models, presenting unadjusted and fully adjusted models, with a random intercept for patient ID. We first calculated descriptive statistics for patients. Following this we calculated Pearson correlations between PM_2.5_, O_3_, and NO_2_ to examine the relationship between the exposures of interest. To make exposures more comparable we then calculate interquartile range (IQR) for use in the models. We controlled for the continuous census block group covariates including average income, percent older housing (built before 1979), percent living in poverty, urbanicity, and percent of the population on public assistance. Demographic covariates included age, sex, and race. As patients were more likely to be sampled from geographic regions closer to hospitals near the flagship UNC Chapel Hill hospital, we control for the climate zones (as identified by the NC Climate Division, map available in Supplementary information) in North Carolina. There are eight climate zones in North Carolina, however due to too few observations we only include zones 3–8 in our analyses (*n* = 15 patients removed).

We calculated eGFR_cr_ as a continuous outcome along with serum creatinine pre-transformation as a secondary outcome. 1-year average concentrations for PM_2.5_, O_3_, and NO_2_ were matched to the date the laboratory test for serum creatinine was completed. We then calculated IQRs for each pollutant during the 1-year period to make them more comparable. Our fully adjusted models included age and race, census block group information (median income, % older housing, % poverty, urbanicity, % on public assistance), geographic region, and exposures (PM_2.5_, O_3_, and NO_2_) along with unrestricted natural cubic spline adjustment for long-term temporal variations with the number of splines based on the Aikake information criteria. We present only the results of multipollutant models, information on single pollutant models is available in Supplementary table S3.

We conducted unconditional multiple logistic regression to estimate odds ratios between first indication date of CKD and air quality for 1-year prior to diagnosis comparing our cases and controls (results of conditional are available in Table S4) [29]. The census block group, demographic, and comorbidity covariates included in our multiple logistic regression models were the same as those included in the linear mixed models. All analyses and visualizations were completed using SAS software version 9.4 and RStudio 4.0.3 [30, 31]. In RStudio we used the package ‘matchit’ for matching cases and controls for our multiple logistic regression models [32].

### Sensitivity analysis

Body mass index (BMI) and smoking status were not reported for all patients in the random sample and were used in secondary analyses to ensure that their inclusion did not alter the linear mixed models described previously. BMI is available for *n* = 18,639 (*n* = 4,834 patients) serum creatinine lab measures and smoking status is available for *n* = 30,913 (*n* = 5,532 patients). Smoking status was separated into five categories including current, current/former, former, never/former, and never. Smoking status was attached to the same day serum creatinine tests were taken, or if it was not assessed that day, then the nearest prior date where smoking status was available. We ran two additional fully adjusted models with the same covariates in addition to BMI (continuous) and smoking status. For both analyses including BMI and smoking status, we calculate associations with and without the additional confounder to ensure that differences seen are not driven by underlying characteristics of the new sampling frames. We ran the fully adjusted models comparing cases of CKD to the entire random sample (available in Supplementary data). For patients without CKD, we ran two models, attaching an exposure date as both first appearance in the hospital system and median visit to ensure that results were consistent at different time points. Lastly, we include only those with street-level geocoded addresses (some patients were coded at zip code) to ensure the most accurate assignment of exposures.

### Stratified analysis

We also stratified by individuals who were exposed to 1-year PM_2.5_ averages ≥ 12 µg/m^3^ and those < 12 µg/m^3^ [33]. This threshold was chosen based on current (2022) National Ambient Air Quality Standards (NAAQS) primary standard for PM_2.5_. To estimate associations of air pollution on patients with low functioning kidneys we stratify by those patients with a measure of an eGFR_cr_ < 60 mL/min/1.73m^2^.

## Results

There were *N* = 7,722 patients with available serum creatinine to calculate eGFR_cr_ and exposure data linked to primary address. Within this group there were *N* = 44,486 serum creatinine tests available during our study period (Tables 1 and 2). BMI is available for *n* = 18,639 (*n* = 4,834 patients) serum creatinine lab measures and smoking status is available for *n* = 30,913 (*n* = 5,532 patients). Patients with serum creatinine measures average 53.6 (SD: 17.9) years of age, are majority female (58.2%), and majority Caucasian (64.7%). The prevalence of diabetes and hypertension in this group were 25.6% and 57.6% respectively. This group was, on average, exposed to 1-year median concentrations at 9.52 (IQR: 1.57) of PM_2.5_ µg/m^3^, 39.7 ppb O_3_ (IQR: 3.21), and 12.8 ppb NO_2_ (IQR: 8.68).

**Table 1:** Descriptive characteristics for the subset of NC-CARES with (1) available eGFR_cr_ values and (2) cases and controls for patients diagnosed with CKD

**Patients** **with** **eGFR**_**cr**_ ***N* = 7,722**			
**Characteristics**	**n (%)**		
Female	4,498 (58.2)		
Male	3,227 (41.8)		
Caucasian	5,000 (64.7)		
African American	2,128 (27.6)		
Other	596 (7.72)		
Diabetes	1,976 (25.6)		
Hypertension	4,453 (57.6)		
**Mean (SD or %)**			
Age	53.6 (17.9)		
Education	36.3 (24.8)		
Older housing	40.6 (23.3)		
Income (USD)	58,005.9 (29,470.8)		
Median house value (USD)	203,462.7 (119,096.6)		
Poverty (%)	16.6 (13.8)		
Public assistance (%)	1.99 (3.05)		
Urbanicity	62.9 (41.8)		
**Median (IQR)**			
PM_2.5_ (µg/m^3^)	9.52 (1.57)	NO_2_ (ppb)	12.8 (8.68)
O_3_ (ppb)	39.7 (3.21)		
**Patients with CKD and never-diagnosed *N* = 4,952**			
	Non-CKD	CKD	Total
**n (%)**			
Female	2,199 (55.9)	553 (54.5)	2,752
Male	1,738 (44.2)	462 (45.5)	2,200
Caucasian	2,279 (57.9)	560 (55.2)	2,839
African American	1,419 (36.0)	415 (40.9)	1,834
Other Race	239 (6.1)	40 (3.9)	279
Diabetes	1,049 (26.6)	476 (46.9)	1,525
Hypertension	2,422 (61.5)	860 (84.7)	3,282
**Mean (SD)**			
Age	65.4 (16.4)	65.3 (16.4)	65.4 (16.4)
Area level covariates*			
Income	58,199.3 (27,147.3)	54,710.5 (25,511.6)	57,484.2 (26,854.7)
Poverty (%)	16.1 (12.6)	17.6 (13.9)	16.41 (12.9)
Urbanicity	66.7 (40.7)	60.9 (42.1)	65.47 (41.1)
Public assistance (%)	1.7 (2.7)	2.0 (2.9)	1.75 (2.8)
Median house value	211,306.8 (120,322.1)	197,977.3 (117,589.7)	208,574.7 (119,876.2)
Education	38.2 (25.47)	35.0 (24.8)	37.5 (25.4)
**Median (IQR)**			
PM_2.5_ (µg/m^3^)	11.31 (3.33)	11.18 (3.64)	11.27 (3.39)
O_3_ (ppb)	41.66 (3.35)	41.41 (3.39)	41.59 (3.37)
NO_2_ (ppb)	15.44 (10.77)	14.11 (10.82)	15.10 (10.80)

^*^Data for block groups come from the 2010 US Census/2013 5-Year ACS; any category that does not sum to 100% is a result of rounding; Education is measure by percentage with a bachelor’s degree or higher; older housing refers to the percentage of houses built before 1979

**Table 2:** Pearson correlations of PM_2.5_, O_3_, and NO_2_ for both the linear mixed and the multiple logistic regression models

**Linear mixed models**			
	PM_2.5_	O_3_	NO_2_
PM_2.5_	1.00	0.53	0.40
O_3_	-	1.00	0.11
NO_2_	-	-	1.00
**Multiple logistic regression models**			
	PM2.5	O3	NO2
PM_2.5_	1.00	0.55	0.38
O_3_	-	1.00	0.2
NO_2_	-	-	1.00

We included *N* = 4,952 patients in our case–control sample that captures all patients identified with CKD and corresponding controls. Within this group, we identified 1,015 patients with severely limited kidney function (ICD code or two eGFR_cr_ < 60 mL/min per 1.73 m^2^), and 3,937 non-CKD patients as controls. Those with CKD were more likely to be diagnosed with diabetes and/or hypertension. Patients with CKD had patterns of lower block group SES status as indicated by higher percent poverty, lower average income, and median house value. However, there were few differences between block-level percentage poverty or those on public assistance. Those diagnosed with CKD were exposed to lower 1-year median concentrations of PM_2.5_ (11.2 µg/m^3^ IQR: 3.64), O_3_ (41.4 ppb IQR: 3.39), and NO_2_ (14.1 ppb IQR: 10.8) when compared to the non-CKD patients median PM_2.5_ 11.3 µg/m^3^ (IQR: 3.33), O_3_ 41.7 ppb (IQR: 3.35), and NO_2_ 15.4 ppb (IQR: 10.8). Standardized mean difference (SMD) in propensity scores for diagnostic date vs. hospital visit dates between cases and controls were 0.048 on average (full table of summary balances for matched data available in Supplementary table S2). All our SMDs between cases and control were less than 0.1, indicating adequate balancing, with the exception of African American patients (SMD = 0.11) and Other Race (SMD = -0.11) and may limit the interpretability of the results in these cases.

**Fig. 1:**
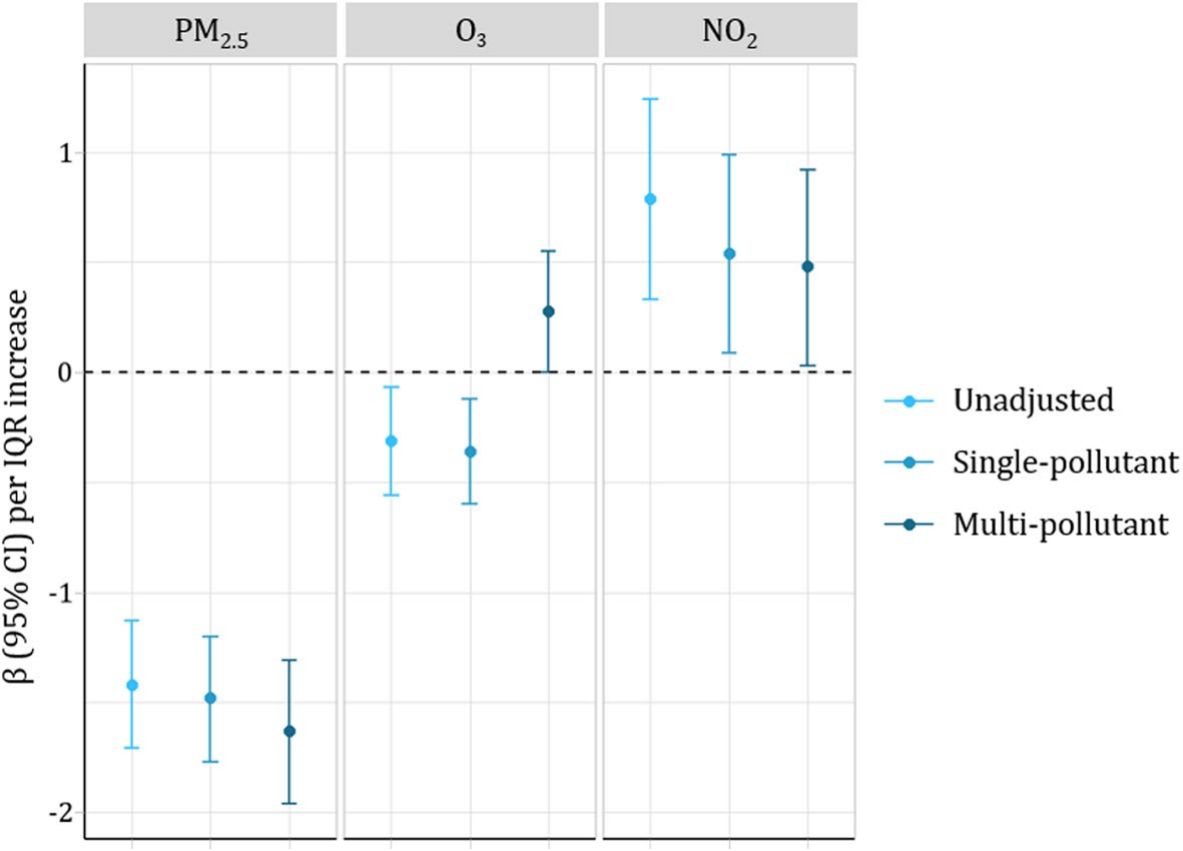
Results of linear mixed models examining the associations of air pollutants with eGFR_cr_

Results from multiple linear regression models estimate an association between IQR increases in PM_2.5_and a decline in eGFR_cr_ (-1.63 mL/min/1.73m^2^, 95% CI: -1.96, -1.31). The results for O_3_ and NO_2_ are 0.28 mL/min/1.73m^2^ (95% CI: 0.00 0.55) and 0.48 (95% CI: 0.03, 0.92), respectively. Likewise, there was a positive association between PM_2.5_ and serum creatinine (0.052 mg/dL, 95% CI: 0.031, 0.073). We observed an inverse association between O_3_ and serum creatinine and no association between NO_2_ and serum creatinine, with estimates of -0.030 (95% CI: -0.048, -0.012) and 0.024 (95% CI: -0.005, 0.052), respectively (Table 3 and Fig. 1).

**Table 3:** Results from mixed linear models & logistic regression of 1-year PM_2.5_, O_3_, NO_2_ and kidney function among a random sample of NC CARES serum creatinine laboratory results (*N* = 44,486)

	IQR 1-year PM_2.5_ (µg/m^3^)	IQR 1-year O_3_ (ppb)	IQR 1-year NO_2_ (ppb)
eGFR_cr_ (mL/min/1.73m^2^) β (95% CI)			
Model 1	-1.44 (-1.72, -1.15)	-0.32 (-0.56, -0.08)	0.80 (0.34, 1.25)
Model 2	-1.63 (-1.96, -1.31)	0.28 (0.00 0.55)	0.48 (0.03, 0.92)
CKD aOR (95% CI)			
Model 1	0.87 (0.73, 1.05)	0.91 (0.84, 1.00)	0.73 (0.66, 0.80)
Model 2	1.04 (0.88, 1.22)	0.89 (0.78, 1.01)	0.79 (0.68, 0.92)

^a^Model 1 Estimate with only random intercepts for patients and spline adjustment for temporal variation

^b^Model 2 fully adjusted linear mixed model estimate for temporal variations, age, sex, race, comorbidities, and census block group

^c^IQR for exposures in our eGFRcr analysis are: PM_2.5_ – 1.43 µg/m^3^; O_3_ – 2.81 ppb; NO_2_ – 8.49 ppb

^d^IQR for exposures in our CKD analysis are: PM_2.5_ – 3.39 µg/m^3^; O_3_ – 3.36 ppb; NO_2_ – 10.45 ppb

**Fig. 2:**
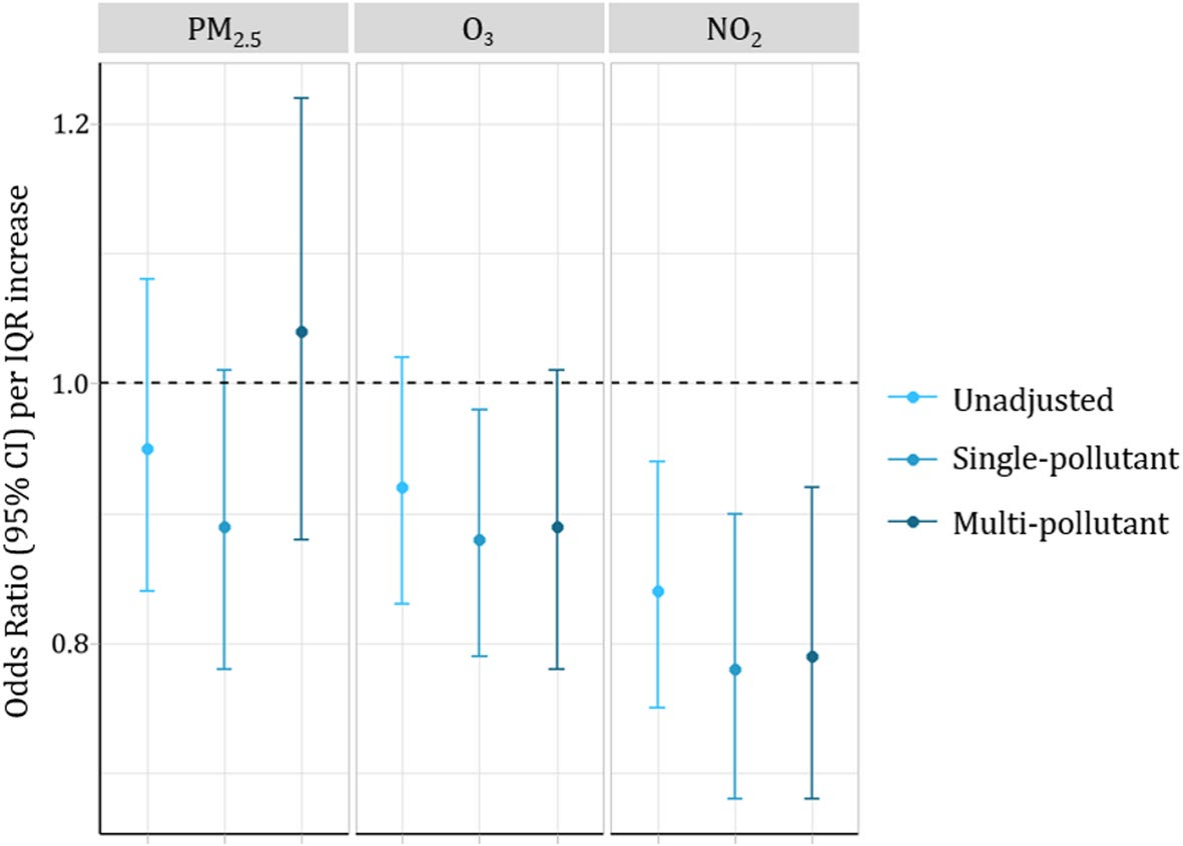
Results of logistic regression models examining the associations of air pollutants with CKD

In the fully adjusted logistic regression model, neither PM_2.5_ or O_3_ were associated with CKD with aORs of 1.04 (95% CI: 0.88, 1.22) and 0.89 (95% CI: 0.78, 1.01) respectively. NO_2_ was inversely associated with CKD with an aOR of 0.79 (95% CI: 0.68, 0.92 (Fig. 2).

For patients with an eGFR_cr_ ≥ 60 mL/min/1.73 m^2^ the association between IQR increases in PM_2.5_ and eGFR_cr_ was -1.06 (95% CI: -1.34, -0.77). For those with impaired renal function, < 60 mL/min/1.73 m^2^, the association was weaker, -0.51 (95% CI: -0.90, -0.13). For those exposed to less than 12 µg/m^3^ of PM_2.5_ the associated with eGFR_cr_ were weaker, while at concentrations above 12 µg/m^3^ the associations were stronger at -0.78 (95% CI: -1.13, -0.43) and -2.58 (95% CI: -3.82, -1.33). When BMI was included in our fully adjusted model, the association of PM_2.5_ with eGFR_cr_ was -0.74 (95% CI: -1.21, -0.28). In the second model with smoking status included, the association of PM_2.5_ with eGFR_cr_ were -1.00 (95% CI: -1.30, -0.70) (note, we did not include both BMI and smoking status in the same model). To ensure that our models including smoking status or BMI cohorts were not altering our general results we ran these two groups without the additional covariates. The results of our models without including the confounder were -1.01 (95% CI: -1.31, -0.71) for smoking status and -0.74 (95% CI: -1.20, -0.28) for BMI.

PM_2.5_ was more strongly associated with reduced renal function in African American patients (-2.40, 95% CI: -3.00, -1.79). While Caucasian patients (-1.27, 95% CI: -1.66, -0.88) showed weaker association between PM_2.5_ and renal function than the main model. The association between PM_2.5_ and eGFR_cr_ was -1.85 (95% CI: -2.32, -1.39 for men and -1.31 (95% CI: -1.76, -0.87) for women. After restricting to only patients with street-level geocoded addresses, there were 6,710 patients and 40,461 unique tests for serum creatinine left in our sample. For those with street-level geocoded addresses the association between PM_2.5_ and eGFR_cr_ was similar as for the entire patient group with an estimate of -1.57 (95% CI: -1.91, -1.23). For this group both O_3_ and NO_2_ were not associated with eGFR_cr_ (available in Supplementary information).

## Discussion

In this study, we examined the relationship between 1-year average PM_2.5_, O_3_, and NO_2_ concentrations with kidney function as measured by eGFR_cr_ and first indication of CKD. Only increases in 1-year mean concentrations of PM_2.5_ were associated with a decrease in eGFR_cr_ while both O_3_ and NO_2_ were not associated. The trends seen for eGFR_cr_ were similar to the associations between the three air pollutants and serum creatinine prior to transformation. For first indication of CKD, we observe null associations between 1-year concentrations of PM_2.5_ and O_3_ and inverse association with NO_2_.

These results support other epidemiologic studies that report an inverse association between IQR increases in PM_2.5_ and eGFR_cr_ [34–36]. Likewise, observed positive associations between O_3_ and eGFR_cr_ has been reported in other studies [37]. Weaver et al. report a lack of association between long term O_3_ exposure and decreased eGFR in a manner consistent to these results [28]. These findings contrast to prior studies that find associations between NO_2_ and kidney function as measured by both eGFR and risk of CKD/ESKD [38, 39]. Li et al., 2021 examined a smaller population (*n* = 169) of older adults, while Liang et al. 2021 reported the results of a large (*n* = 47,086) nationally representative sample. It is possible that there is a relationship between NO_2_ and decreased renal function, but 1-year concentrations are not long enough to capture the relationship. NO_2_ is very source dependent, and future work may want to investigate those living near roadways, as this is a major source of NOx exposure in the United States. In a nationwide cross-sectional study in China, Liang et al. show an increased risk of developing CKD with longer term exposure to higher concentrations of NO_2_, with the risk being greatest at 5-years of exposure (for example Liang et al. and Li et al. report median concentrations for NO_2_ at approximately 24 and 23 ppb respectively) [38]. Though, generally, levels of air pollution in China are higher than the US/Europe, so there may be dose-dependent responses we do not see in this study, particularly as these two studies reported higher concentrations of NO_2_.

Despite higher concentrations of PM_2.5_ being associated with lower eGFR_cr_, we do not see a similar relationship between PM_2.5_ and first-time indication of CKD. Inverse associations were seen with O_3_ and NO_2_ with incident CKD. Prior studies, such as Yang et al., 2022 have reported positive association between O_3_ and the prevalence of CKD in a nationwide Chinese study [37]. Similar findings in other studies have found no associations between O_3_ and incidence of CKD, suggesting that more studies are needed that reflect impacts on the general population [40]. O_3_ is relatively less studied than other criteria air pollutants in association with CKD [41]. It is notable that the majority of studies focusing on air pollution and kidney function/disease take place in either the United States or Asia or studied a special population such as military veterans [13, 35]. As such, more research should be conducted to better understand these associations in other geographic regions, cultural and social context, climatic regions, etc.

African American patients were exposed to higher concentrations of PM_2.5_ on average than both Caucasian and patients of another race. In our stratified analyses, African American patients were more likely to have lower eGFR_cr_ when compared to Caucasians. These differences in outcome by race are likely a result of social determinants of health impacting disparities in renal health [42]. The African American population in the US, relative to other ethnicities, make up a disproportionately large percentage of those with CKD [43]. It is important for future research to investigate other potential environmental, social contributors, and interactions between these to these health disparities.

We further conducted stratified analyses by PM_2.5_ concentrations, age, and limited analyses to those with street-level geocoded addresses. We found the associations between PM_2.5_ and decreased in eGFR_cr_ were decreased at concentrations < 12 µg/m^3^ but increased at higher levels. This particular result needs further investigation as these findings do not directly support the growing body of evidence that even at lower concentrations (e.g., US NAAQS standards), air pollution has deleterious impacts on health [44–46]. On the impacts of age, the estimate for PM_2.5_ and eGFR_cr_ was stronger for those < 65 years of age, with weaker associations observed for older patients. This may be a result of older patients being more likely to be on anti-hypertensive medication or medication for diabetes. After stratifying by CKD status, or those not identified with CKD, the association with O_3_ is inverse. For individuals diagnosed with CKD this may be that, given the high percentage of comorbidities, the impact of air pollution may be exacerbated by directly impacting regulation of both blood sugar and pressure [47].

A limitation of this study is that the patients included in these analyses resided predominantly in central North Carolina, where the majority of UNCHCS affiliated hospitals or clinics are located. Due to relative underrepresentation our African American and Other Race patients were not matched to the level of our Caucasian patients, which could introduce bias. With address geocoding there is always the possibility of misclassification that cannot be assumed to trend towards the null. As such this study may lack generalizability to the general population. Using 1-year average air pollution concentrations does not capture the entirety of the time air pollution potentially impacted kidney health. Census block group level covariates do not necessarily capture individual SES, which ideally would have been at the individual level; there could be residual confounding by SES. Finally, it is likely that serum creatinine was measured more often for patients with suspected renal dysfunction, biasing the sample towards those with already impaired kidney function. Unexpected directionality arose concerning associations between NO_2_ and kidney function in our analyses. This is likely due to additional, unmeasured confounding, dose-dependent effects, geographic proximity to roadways, etc. that were not addressed in the scope of this work. This is an area we recommend additional research be focused. Further, there is the possibility that there is the possibility that there is a time x exposure interaction that was not accounted for in this current study. Lastly there are limitations when using EHR data such as representativeness, the data available, missing or incorrectly entered measures, etc.

One of the strengths of this study is that it takes a random sample of patients visiting the North Carolina healthcare system and does not focus on a special sub-population. This random sample has near complete clinical phenotyping and well validating air pollution modeling estimates, matched with high precision geocoding. Additionally, by utilizing e-phenotyping of CKD, we may be more accurately estimating associations between air pollution and reduced renal function as CKD is often not diagnosed until the latter stages.

In conclusion, we observed reduced renal function, as measured by eGFR_cr_, with 1-year concentrations on PM_2.5_, but not with O_3_ or NO_2_. No exposures were associated with increased odds of CKD, while NO_2_ was inversely associated. This study provides further evidence that long-term exposure to fine particulate matter is associated to reduced renal function and may contribute to adverse outcomes.

### Supplementary Information

**Supplementary Material 1. **

## Data Availability

The data for this study contain identifiable information and cannot be shared. More information regarding data access and requests for access may be found here https://tracs.unc.edu/. Analytic code used in this publication are available from the authors upon reasonable request.

## Abbreviations

BMI: Body Mass Index
CKD: Chronic Kidney Disease
CMAQ: Community Multiscale Air Quality Modeling System
EHRs: Electronic Health Record
EPA CARES: Environmental Protection Agency’s Clinical and Archived Records Research for Environmental Studies
ESKD: End-Stage Kidney Disease
eGFR: Estimated Glomerular Filtration Rate
eGFR_cr_: Serum Creatinine Estimated Glomerular Filtration Rate
FIPS: Federal Information Processing System
IQR: Interquartile range
NAAQS: National Ambient Air Quality Standards
NKDEP: National Kidney Disease Education Program
NO_2_: Nitrogen dioxide
O_3_: Ozone
PM_2.5_: Particulate matter < 2.5 µm in diameter
Scr: Serum Creatinine
SES: Socioeconomic Status
UA: Urinary Albumin
UACR: Urinary Albumin/creatinine ratio
