# Supplementary material for: Associations between long-term exposure to air pollution and kidney function utilizing electronic healthcare records: a cross-sectional study
Source: Environ Health. 2024 Apr 23;23:43. doi: 10.1186/s12940-024-01080-4 (PMC11036746; doi:10.1186/s12940-024-01080-4)

**Supplementary information**

**Table S1.** ICD-9 and ICD-10 codes used for the identification of physician diagnosed CKD and comorbidities

| Disease | ICD-Code |
| --- | --- |
| Chronic kidney disease | ICD9- 585.3, 585.4, 585.5, 585.6  ICD10- N18.3, N18.4, N18.5, N18.6 |
| Hypertension | ICD9- 401.9  ICD10- I10 |
| Diabetes | ICD9- 250.0, 250.00, 250.02, 250.1, 250.10, 250.12, 250.2, 250.20, 250.22, 250.3, 250.30, 250.32, 250.4, 250.40, 250.42, 250.5, 250.50, 250.52, 250.6, 250.60, 250.62, 250.7, 250.70, 250.72, 250.8, 250.80, 250.82, 250.9, 250.90, 250.92  ICD10- E11.0 – E11.9 |

**Table S2.** Summary of balance for matched cases, diagnosed by ICD code or e-phenotype, and controls

|  | Means case | Means control | Std mean difference | Var ratio | eCDF mean | eCDF max | Pair distance |
| --- | --- | --- | --- | --- | --- | --- | --- |
| Distance | 0.03 | 0.03 | 0.01 | 0.99 | 0.01 | 0.02 | 0.02 |
| Age | 65.32 | 65.63 | -0.02 | 1.06 | 0.01 | 0.03 | 0.69 |
| Date | 14,493.34 | 14,429.13 | 0.048 | 1.14 | 0.03 | 0.05 | 1.06 |
| Female | 0.54 | 0.56 | -0.03 | . | 0.02 | 0.02 | 0.96 |
| Male | 0.46 | 0.44 | 0.03 | . | 0.02 | 0.02 | 0.96 |
| Black | 0.41 | 0.36 | 0.11 | . | 0.05 | 0.05 | 0.82 |
| Other | 0.04 | 0.06 | -0.11 | . | 0.02 | 0.02 | 0.48 |
| White | 0.55 | 0.58 | -0.06 | . | 0.03 | 0.03 | 0.89 |
|  | All | Matched | Unmatched | Discarded |  |  |  |
| Control | 99,754 | 4,084 | 95,670 | 0 | . | . | . |
| Case | 1,021 | 1,021 | 0 | 0 | . | . | . |

*Standardized mean differences < 0.1 indicate adequate balance

**Table S3.** Results of fully adjusted single pollutant (1) linear mixed and (2) logistic regression models for eGFRcr and diagnosis of CKD

| Results of single pollutant linear mixed model | | | |
| --- | --- | --- | --- |
|  | IQR 1-year PM_2.5_ (µg/m^3^) | IQR 1-year O_3_  (ppb) | IQR 1-year NO_2_  (ppb) |
| Beta (95% CI) | -1.47  (-1.75, -1.18) | -0.35  (-0.59, -0.11) | 0.58  (0.13, 1.03) |
| Results of single pollutant logistic regression | | | |
|  | IQR 1-year PM_2.5_ (µg/m^3^) | IQR 1-year O_3_  (ppb) | IQR 1-year NO_2_  (ppb) |
| aOR (95% CI) | 0.89  (0.78, 1.01) | 0.88  (0.79, 0.98) | 0.78  (0.68, 0.90) |

**Table S4.** Results of logistic regression models comparing patients with CKD to the entire random sample cohort using 1-year average concentrations for both first visit and median visit as exposures

|  | IQR year PM_2.5_ (µg/m^3^) | IQR 1-year O_3_  (ppb) | IQR 1-year NO_2_  (ppb) |
| --- | --- | --- | --- |
| First visit in the UNC hospital system | | | |
| Model 1 | 1.05  (0.91, 1.22) | 2.00  (1.84, 2.17) | 1.03  (0.94, 1.13) |
| Model 2 | 1.05  (0.89, 1.23) | 2.16  (1.97, 2.37) | 1.2  (1.06, 1.37) |
| Median visit in the UNC hospital system | | | |
| Model 1 | 1.00  (0.86, 1.17) | 2.04  (1.88, 2.22) | 1.03  (0.93, 1.13) |
| Model 2 | 1.03  (0.87, 1.22) | 2.20  (2.00, 2.42) | 1.20  (1.05, 1.36) |
| Median visit in the UNC hospital system from fully adjusted conditional regression model | | | |
| Model 2 | 2.56  (1.97, 3.39) | 0.84  (0.73, 0.97) | 0.83  (0.71, 0.98) |

*Model 1 presents unadjusted estimates and Model 2 presents the results of the fully adjusted logistic regression models

**Table S5**. Results of fully adjusted models for stratified and sensitivity analysis examining associations between air pollutants and eGFR_cr_

|  | IQR year PM_2.5_ (µg/m^3^) | IQR 1-year O_3_  (ppb) | IQR 1-year NO_2_  (ppb) |
| --- | --- | --- | --- |
| Stratified analyses | | | |
| Female | -1.31  (-1.76, -0.87) | 0.20  (-0.18, 0.58) | -0.09  (-0.72, 0.55) |
| Male | -1.85  (-2.32, -1.39) | 0.26  (-0.13, 0.66) | 1.04  (0.42, 1.66) |
| ≥ 12 PM_2.5_ µg/m^3^ | -2.58  (-3.83, -1.33) | 0.86  (0.00, 1.71) | 0.03  (-0.90, 0.97) |
| < 12 PM_2.5_ µg/m^3^ | -078  (-1.13, -0.43) | 0.78  (0.49, 1.08) | 0.61  (0.09, 1.12) |
| ≥ 60 eGFR_cr_ mL/min/1.73 m^2^ | -1.06  (-1.34, -0.77) | 0.08  (-0.17, 0.33) | 0.37  (-0.03, 0.77) |
| < 60 eGFR_cr_ mL/min/1.73 m^2^ | -0.51  (-0.90, -0.13) | 0.36  (0.03, 0.69) | 0.23  (-0.28, 0.74) |
| Never diagnosed with CKD | -0.92  (-1.28, -0.56) | -0.27  (-0.58, 0.04) | 0.82  (0.33, 1.30) |
| Smoking status | -1.01  (-1.31, -0.71) | 0.49  (0.27, 0.71) | -0.08  (-0.63, 0.46) |
| Caucasian | -1.27  (-1.66, -0.88) | -0.12  (-0.46, 0.21) | 1.09  (0.54, 1.63) |
| African American | -2.40  (-3.00, -1.79) | 0.99  (0.49, 1.50) | -0.93  (-1.77, -0.10) |
| Other race | -1.36  (-2.80, 0.07) | 0.12  (-1.07, 1.32) | 0.09  (-1.86, 2.04) |
| BMI known | -0.74  (-1.20, -0.28) | 0.18  (-0.18, 0.53) | 0.40  (-0.32, 1.13) |
| Sensitivity analysis | | | |
| Geocoded Address | -1.57  (-1.91, -1.23) | 0.21  (-08, 0.50) | 0.45  (-0.03, 0.93) |

| Climate Zone | Control | CKD | Total |
| --- | --- | --- | --- |
|  | n (%) | | n |
| Zone 1 | 10 (0.20) | 1 (0.02) | 11 |
| Zone 2 | 0 (0.0) | 4 (0.08) | 4 |
| Zone 3 | 1,624 (41.25) | 490 (48.28) | 2,114 |
| Zone 4 | 1,315 (33.40) | 302 (29.75) | 1,617 |
| Zone 5 | 79 (2.01) | 24 (2.63) | 103 |
| Zone 6 | 593 (15.06) | 126 (12.41) | 719 |
| Zone 7 | 159 (4.04) | 47 (4.63) | 206 |
| Zone 8 | 157 (3.99) | 21 (2.07) | 178 |
| Total | 3,937 | 1,015 | 4,952 |

**Table S6.** Geographic distribution of CKD cases and matched controls over the eight climatic divisions of North Carolina

**Figure S1.** Balance statistics of standardized mean differences pre- and post-matching for CKD cases and controls


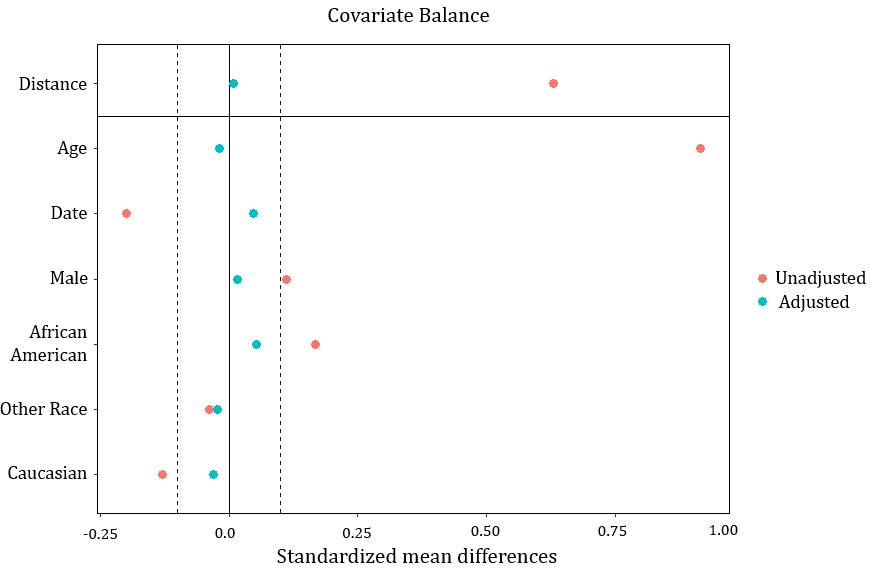


**Figure S2.** Q-Q Plots of pre- and post-matching


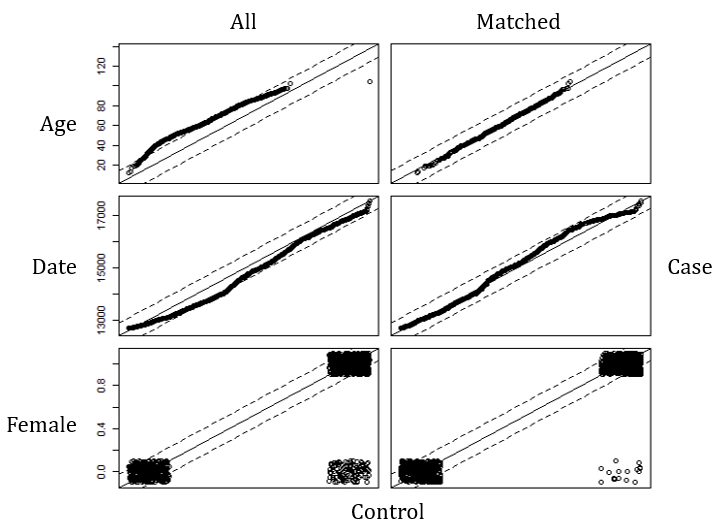


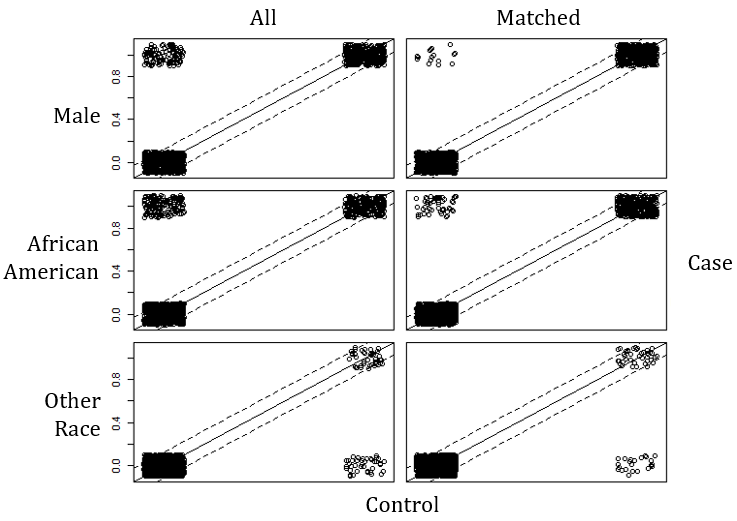


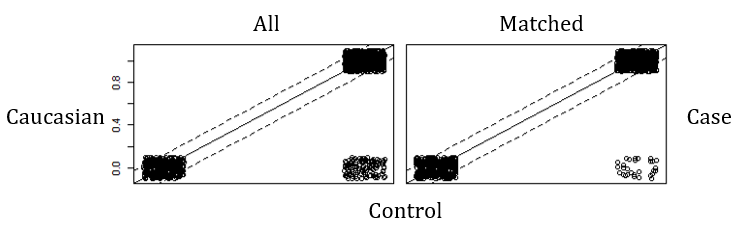


**Figure S3.** Map of the eight climate zones in North Carolina (this image was taken from http://www.climatechange.nc.gov/Climate_Maps_NC.pdf)


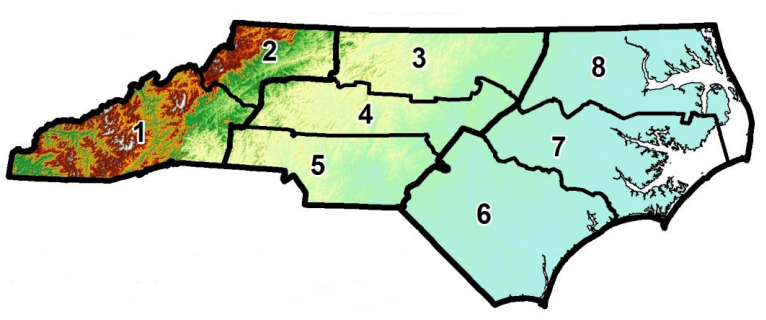

Supplement: Supplementary file 1 — Supplementary Material 1. [file 12940_2024_1080_MOESM1_ESM.docx]
